# TNF-α Affects Human Cortical Neural Progenitor Cell Differentiation through the Autocrine Secretion of Leukemia Inhibitory Factor

**DOI:** 10.1371/journal.pone.0050783

**Published:** 2012-12-07

**Authors:** Xiqian Lan, Qiang Chen, Yongxiang Wang, Beibei Jia, Lijun Sun, Jialin Zheng, Hui Peng

**Affiliations:** 1 Laboratory of Neuroimmunology and Regenerative Therapy, University of Nebraska Medical Center, Omaha, Nebraska, United States of America; 2 Department of Pharmacology and Experimental Neuroscience, University of Nebraska Medical Center, Omaha, Nebraska, United States of America; 3 Department of Pathology and Microbiology, University of Nebraska Medical Center, Omaha, Nebraska, United States of America; University of North Dakota, United States of America

## Abstract

Proinflammatory cytokine tumor necrosis factor-alpha (TNF-α) is a crucial effector of immune responses in the brain that participates in the pathogenesis of several acute and chronic neurodegenerative disorders. Accumulating evidence has suggested that TNF-α negatively regulates embryonic and adult neurogenesis. However, the effect of TNF-α on cell fate decision in human neural progenitor cells (NPCs) has rarely been studied. Our previous studies have shown that recombinant TNF-α enhances astrogliogenesis and inhibits neurogenesis of human NPCs through the STAT3 (signal transducer and activator of transcription 3) pathway. In the current study, we further elucidated the specific mechanism involved in TNF-α-induced astrogliogenesis. We found that TNF-α activated STAT3 at delayed time points (6 h and 24 h), whereas conditioned medium collected from TNF-α-treated NPCs induced an immediate STAT3 activation. These data suggest TNF-α plays an indirect role on STAT3 activation and the subsequent NPC differentiation. Further, we showed that TNF-α induced abundant amounts of the IL-6 family cytokines, including Leukemia inhibitory factor (LIF) and Interleukin 6 (IL-6), in human NPCs. TNF-α-induced STAT3 phosphorylation and astrogliogenesis were abrogated by the addition of neutralizing antibody for LIF, but not for IL-6, revealing a critical role of autocrine secretion of LIF in TNF-α-induced STAT3 activation and astrogliogenesis. This study generates important data elucidating the role of TNF-α in neurogenesis and may provide insight into new therapeutic strategies for brain inflammation.

## Introduction

Neural stem/progenitor cells (NSPCs) are self-renewing, multipotent cells that are capable of differentiating into neurons, astrocytes, and oligodendrocytes [1]–[3]. Localized within specific neurogenic regions of the developing and adult brain, these cells contribute to brain patterning, memory formation, and brain repair [4]–[8]. NPCs are activated during neuroinflammatory conditions and preferentially migrate to and differentiate at the site of injury, relying in part on soluble mediators released by damaged tissue and activated immune cells [5], [9], [10]. In particular, inflammatory cytokines, abundantly secreted following brain injury or infection, contribute to the microenvironment of neurogenic niches where neural precursor cells reside and influence neurogenesis.

Among these proinflammatory mediators, tumor necrosis factor (TNF)-α is an important effector of immune response in the brain and is involved in the pathogenesis of several acute and chronic neurodegenerative disorders, such as Parkinson's disease, Multiple Sclerosis, and stroke [11]. TNF-α has been shown to negatively affect embryonic and adult neurogenesis through inhibition of NPC proliferation [12]–[15]. However, there have been relatively few studies examining the effect of TNF-α on cell fate decision. Using embryonic rat whole-brain neurosphere cultures, Liu *et al.* have demonstrated that TNF-α reduced the number of NPCs adopting a neuronal phenotype under differentiating conditions [13]. Exposure of hippocampal NPCs to TNF-α has been shown to have a detrimental effect on their neuronal lineage fate through increased expression of Hes1 [14]. However, exposure of NPCs derived from the SVZ of neonatal mice to TNF-α has also been reported to induce differentiation to a neuronal phenotype [16]. Little information is known about how TNF-α regulates human NPC differentiation. Previous studies in our laboratory have demonstrated that TNF-α released from HIV-1-infected and immune-activated macrophages increased astrocytic differentiation (astrogliogenesis) and inhibited neuronal differentiation of human NPCs [17], [18]. However, the molecular mechanism of this tilted NPC differentiation remains unclear.

In the current study, we used human cortical NPC cultures to investigate the mechanism involved in TNF-α-mediated NPC differentiation. We found that TNF-α-induced STAT3 activation and subsequent astrogliogenesis is dependent upon the autocrine secretion of LIF.

## Materials and Methods

### Neural progenitor cell culture and conditioned medium

Human fetal brain tissue (12–16 weeks post-conception) was obtained from elective abortions carried out by the University of Washington in full compliance with the University of Washington, the University of Nebraska Medical Center, and the National Institutes of Health (NIH) ethical guidelines, with human subjects Institutional Review Board (IRB) approval no. 96-1826-A07 (University of Washington) and no. 123-02-FB (University of Nebraska Medical Center). A written informed consent is obtained by the University of Washington using an IRB approved consent form. Human cortical NPCs were isolated as previously described [19]. NPCs were cultured in substrate-free tissue culture flasks and grown as spheres in neurosphere initiation medium (NPIM), which consists of X-Vivo 15 (BioWhittaker, Walkersville, ME) with N2 supplement (Gibco BRL, Carlsbad, CA), neural cell survival factor-1 (NSF-1, Bio Whittaker), basic fibroblast growth factor (bFGF, 20 ng/ml, Sigma-Aldrich, St. Louis, MO), epidermal growth factor (EGF, 20 ng/ml, Sigma-Aldrich), leukemia inhibitory factor (LIF, 10 ng/ml, Chemicon, Temecula, CA), and N-acetylcysteine (60 ng/ml, Sigma-Aldrich). Cells were passaged at two-week intervals as previously described [19].

To collect conditioned medium, dissociated NPCs were plated on poly-D-lysine-coated cell culture dishes in NPIM for 24 h. Cells were rinsed with fresh X-Vivo 15 and then treated with TNF-α (20 ng/ml) in X-Vivo 15 for 24 h. The NPC conditioned medium (NCM) was then harvested, cleared of free-floating cells by centrifugation for 5 min at 1200 rpm, and stored at −80°C.

To block the soluble factors in NCM, it was pre-incubated with neutralizing antibodies for LIF (1 µg/ml, R&D Systems, Minneapolis, MN) or IL-6 (1 µg/ml, R&D Systems) for 1 h at 37°C. Cells were then treated with NCM with or without neutralizing antibodies for 30 min. Whole-cell protein lysates were collected for Western blot or cells were fixed for immunocytochemical analysis.

### Human neural progenitor cell differentiation

Neuronal differentiation of NPCs was performed as previously described [19]. Briefly, dissociated NPCs were plated on poly-D-lysine-coated cell culture dishes in NPIM for 24 h. Cells were subsequently changed to serum-free Neurobasal medium (Gibco BRL) supplemented with B27 (NB27 medium) (Gibco BRL) with or without TNF-α. For the inhibition of releasing factors in response of TNF-α treatment, cells were pre-incubated with neutralizing antibodies for LIF or IL-6 for 1 h at 37°C and then treated with TNF-α. Cells were collected for protein, or fixed for immunocytochemical staining 6 days after TNF-α treatment.

### Immunocytochemistry

Cells were fixed in 4% PFA and washed in PBS as previously described [19]. Cells were then incubated overnight with primary antibodies, followed by Alexa Fluor secondary antibodies, goat anti-mouse IgG Alexa Fluor 488 and goat anti-rabbit IgG Alexa Fluor 594 (Molecular Probes, Eugene, OR, 1∶800) for 1 h at room temperature. Primary antibodies included mouse anti-β-III-tubulin (Sigma-Aldrich, 1∶400), rabbit anti-GFAP (glial fibrillary acidic protein, Dako, Carpinteria, CA, 1∶1000), mouse anti-nestin (Chemicon, 1∶600), rabbit anti-phospho STAT3 (P-STAT3, Cell Signaling Technologies, 1∶1000), and mouse anti-LIF (R & D Systems, 1∶400). All antibodies were diluted in 0.1% Triton X-100, 2% BSA in PBS. Cells were counterstained with DAPI (Sigma-Aldrich) to identify nuclei. Morphological changes were visualized and captured with a Nikon Eclipse E800 microscope equipped with a digital imaging system. Images were imported into Image-ProPlus, version 7.0 (Media Cybernetics, Sliver Spring, MD) for quantification. Ten to fifteen random fields (total 500–1000 cells per culture) of immunostained cells were manually counted using a 20× objective.

### Western blotting

Cells were rinsed twice with PBS and lysed by M-PER Protein Extraction Buffer (Pierce, Rockford, IL) containing 1× protease inhibitor cocktail (Roche Diagnostics, Indianapolis, IN). Protein concentration was determined using a BCA Protein Assay Kit (Pierce). Proteins (20–30 µg) were separated on a 10% SDS-polyacrylamide gel electrophoresis (PAGE) and then transferred to an Immuno-Blot polyvinylidene fluoride (PVDF) membrane (Bio-Rad, Hercules, CA). After blocking in PBS/Tween (0.1%) with 5% nonfat milk, the membrane was incubated with primary antibodies (phospho- and total-STAT3, Cell Signaling Technologies; β-actin, GFAP, and β-III-tubulin, Sigma-Aldrich) overnight at 4°C followed by horseradish peroxidase-conjugated secondary antibodies (Cell Signaling Technologies, 1∶10,000) and then developed using Enhanced Chemiluminescent (ECL) solution (Pierce). For data quantification the films were scanned with a CanonScan 9950F scanner and the acquired images were then analyzed on a Macintosh computer using the public domain NIH image program (developed at the U.S. National Institutes of Health and available on the internet at http://rsb.info.nih.gov/nih-image/).

### RNA extraction and TaqMan real-time RT-PCR

Total RNA was isolated with TRIzol Reagent (Invitrogen Corp, Carlsbad, CA) and RNeasy Kit (Qiagen Inc., Valencia, CA) according to the manufacture's protocol. Primers used for real-time reverse-transcription polymerase chain reaction (real-time RT-PCR) include IL-6, LIF, Ciliary neurotrophic factor (CNTF) and Glyceraldehyde 3-phosphate dehydrogenase (GAPDH, part # 4310884E, Applied Biosystems Inc). Real-time RT-PCR was carried out using the one-step quantitative TaqMan assay in a StepOne™ Real-Time PCR system (Applied Biosystems Inc.). Relative IL-6, LIF, and CNTF mRNA levels were determined and standardized with a GAPDH internal control using comparative ΔΔCT method. All primers used in the study were tested for amplification efficiencies and the results were similar.

### Enzyme-linked immunosorbent assay (ELISA)

Supernatants were collected for IL-6 and LIF determination by an in house ELISA. Briefly, 96-well micro titer plates (Costar) were coated overnight at room temperature with capture antibodies (R&D Systems) in PBS. Non-specific binding was blocked for 2 h with 1% BSA in PBS. Triplicate samples of cell supernatants or a serial dilution of standards of human recombinant IL-6 or LIF were applied to the wells and incubated overnight at 4°C. Samples were removed and wells were incubated with the biotinylated detection antibodies, followed by 1 h incubation with HRP-conjugated streptavidin (R&D Systems). TMB Substrate Solution (Sigma) was added and the absorbance was determined using a microplate reader (Rio-Rad Laboratories, Hercules, CA) set at 450 nm.

### Statistical analyses

Data were presented as means ± standard deviation (SD) unless otherwise noted. All experiments were repeated at least three times with different donors with triplicate or quadruplicate samples in each assay. All data were evaluated statistically by the analysis of variance (ANOVA), followed by Newman-Keuls multiple comparison tests using software (Prism 4.0, GraphPad Software). In the case of single mean comparison, data were analyzed by t test. *p* values≤0.05 are regarded as statistically significant.

## Results

### TNF-α induces STAT3 activation in human NPCs at delayed time points

**Figure 1:**
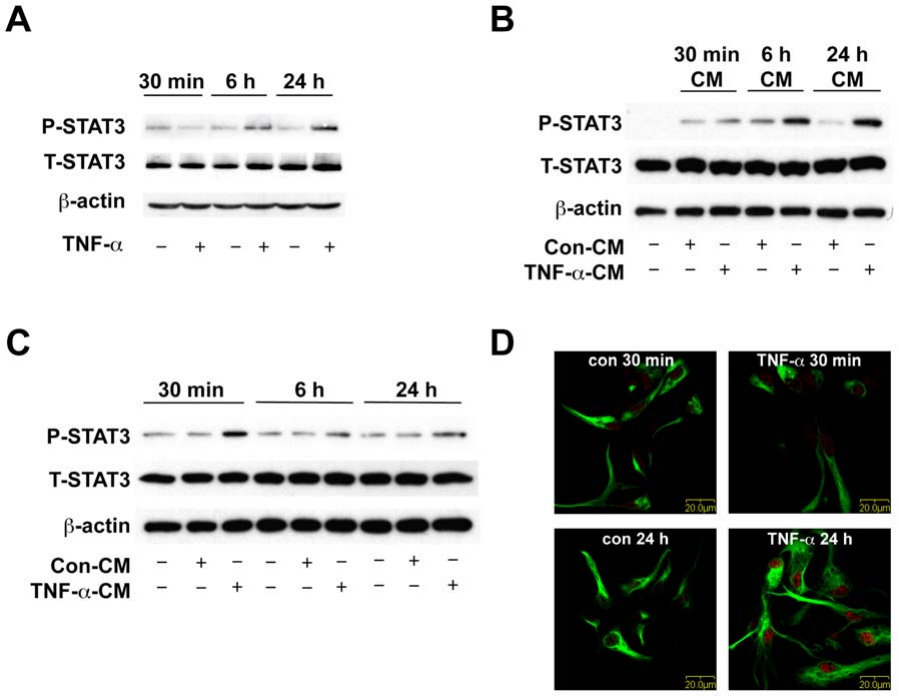
TNF-α induces delayed STAT3 activation in human NPCs. **A**. Human NPCs were treated with 20 ng/ml TNF-α for 30 min, 6 h, and 24 h. Expression of phospho-STAT3 (P-STAT3) and total-STAT3 (T-STAT3) were detected by Western blotting. β-actin was used as a loading control. **B**. Human NPCs were treated with 20 ng/ml TNF-α for 30 min, 6 h, and 24 h. Supernatants were collected as NPC conditioned medium (CM). Parallel cultured human NPCs were treated with control NPC-CM or TNF-α-treated NPC-CM (con-CM or TNF-α-CM) for 30 min. Expression of P-STAT3 and T-STAT3 were detected by Western blotting. β-actin was used as a loading control. **C**. Human NPCs were treated TNF-α-free NPC-CM for 30 min, 6 h, and 24 h. Expression of P-STAT3 and T-STAT3 were detected by Western blotting. β-actin was used as a loading control. **D**. Human NPCs were treated with 20 ng/ml TNF-α for 30 min or 24 h. Cells were immunolabeled with antibodies for the NPC marker Nestin (green) and P-STAT3 (red). Original magnification is ×60 (scale bar 20 µm). Results are representative of three independent experiments.

Previous work in our laboratory has demonstrated that TNF-α increases astrocytic differentiation and inhibits neuronal differentiation of human NPCs. Furthermore, TNF-α induces astrogliogenesis through STAT3 signaling, since siRNA specifically targeting STAT3 (siSTAT3) inhibited TNF-α-induced astrogliogenesis [17], [18]. To elucidate the additional mechanism involved in TNF-α-induced STAT3 activation and subsequent astrogliogenesis, we treated human NPCs with TNF-α and studied STAT3 phosphorylation at different time points (30 min, 6 h, and 24 h) (Figure 1A). TNF-α did not induce immediate STAT3 phosphorylation at 30 min. However, TNF-α induced STAT3 phosphorylation at 6 h and continued to induce even stronger STAT3 phosphorylation at 24 h (Figure 1A).

The delayed STAT3 activation by TNF-α indicates that TNF-α may play an indirect role on STAT3 activation: secreted factors produced by TNF-α-treated NPCs activated the STAT3 pathway at later time points (6 h and 24 h). To test this hypothesis, human NPCs were treated with TNF-α for 30 min, 6 h and 24 h, and supernatants were collected as conditioned medium (CM). Parallel cultured NPCs were then treated with these different time point conditioned media (TNF-α-treated (TNF-α-CM) or control NPC-CM (Con-CM)) for 30 min and cell lysates were collected for Western blot. TNF-α-CM collected at 30 min did not induce a significant increase of STAT3 phosphorylation. In contrast, TNF-α-CM collected at 6 h moderately increased STAT3 phosphorylation; and TNF-α-CM collected at 24 h showed a significant increase of STAT3 phosphorylation as compared with Con-CM treatment (Figure 1B). This result suggests that TNF-α-induced soluble factors, which are highly produced at 24 h, subsequently induce STAT3 phosphorylation in human NPCs in an autocrine manner.

We next studied the kinetics of CM-mediated STAT3 phosphorylation in NPCs. To exclude the effect of residual TNF-α in CM, human NPCs were treated with TNF-α for 6 h, rinsed twice with X-Vivo 15 and then maintained in fresh X-Vivo 15 medium. Twenty-four hours later, the TNF-α-free cell supernatants were collected as TNF-α-free-CM. TNF-α-free-CM treatment induced an immediate STAT3 phosphorylation at 30 min, but not at 6 h or 24 h (Figure 1C). This result suggests that secreted factors produced by TNF-α-treated NPCs have differential kinetics in activating the STAT3 pathway compared to TNF-α.

To further characterize TNF-α-induced STAT3 activation in NPCs, we performed immunocytochemical studies with NPC culture using antibodies against phospho-STAT3 and nestin, a neural progenitor cell marker. Consistent with the Western blot result, TNF-α did not increase STAT3 phosphorylation or nucleus translocation at the early time point (30 min). However, at 24 h following TNF-α treatment, we observed apparent STAT3 phosphorylation and nucleus translocation (Figure 1D). In addition, the active form of STAT3 co-localized with nestin, suggesting phospho-STAT3 signal cascade occurs within the nestin-positive NPC population.

### TNF-α induces IL-6 family cytokine production

**Figure 2:**
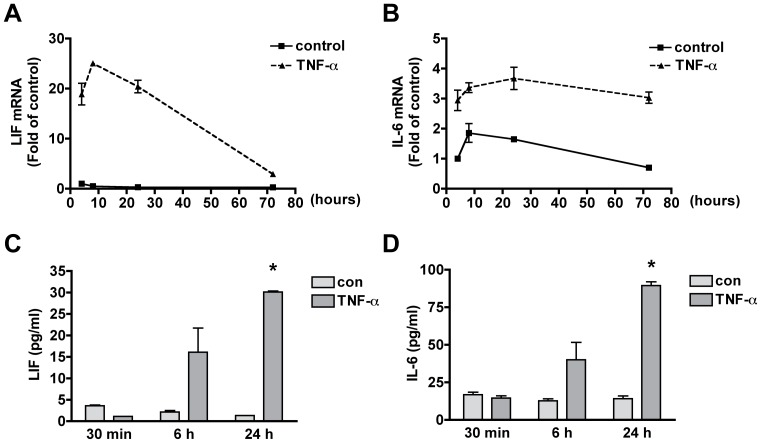
TNF-α increases the expression of IL-6 and LIF in human NPCs. **A–B**. NPCs were treated with 20 ng/mL TNF-α for 4, 8, 24 and 72 h. mRNA expression of IL-6 (A) and LIF (B) were detected by TaqMan Real Time RT-PCR. Data were normalized to GAPDH and presented as fold change compared to control. ** p<0.01 in comparison to control. Results are representative of three independent donors. **C–D**. NPCs were treated with 20 ng/mL TNF-α for 30 min, 6 h and 24 h. The supernatants were assayed for IL-6 and LIF production by ELISA. Data represent the mean ± SD of triplicate samples from a representative result of three donors. * p<0.05 in comparison to control.

Members of the IL-6 cytokine family such as LIF, IL-6 and ciliary neurotrophic factor (CNTF) have been reported to activate the Jak-STAT signaling pathway and promote astroglial differentiation through the gp130-mediated signaling pathway [20], [21]. To identify which IL-6 family cytokines are involved in TNF-α-induced astrogliogenesis, we treated human NPCs with TNF-α (20 ng/ml) for 4, 8, 24, and 72 h and analyzed the mRNA expression of IL-6, LIF and CNTF using real time RT-PCR. IL-6, LIF and CNTF were all expressed in human NPCs. However, TNF-α specifically increased the mRNA expression of LIF and IL-6 in a time dependent manner (Figure 2A, B), but not CNTF (data not shown). We also detected LIF and IL-6 protein levels in TNF-α-treated NPC supernatant by ELISA. TNF-α modestly increased IL-6 and LIF production at 6 h, and significantly increased IL-6 and LIF production at 24 h, but not at 30 min (Figure 2C, D). These data indicate that TNF-α induces IL-6 and LIF production via transcriptional regulation, but not through direct secretion.

**Figure 3:**
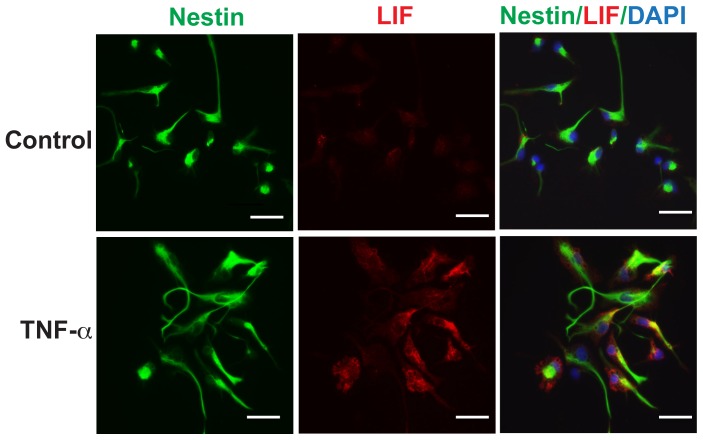
TNF-α induces LIF in human NPCs. NPCs were treated with 20 ng/mL TNF-α for 14 h. Cells were immunolabeled with antibodies to NPC maker nestin (green) and LIF (red). Nuclei were stained with DAPI (blue). Original magnification is x 20 (scale bar 10 µm). Results are representative of two independent experiments.

To confirm that LIF is produced by human NPCs, we further assess the protein levels of LIF expression by immunocytochemistry. Human NPCs were treated with TNF-α (20 ng/ml) for 14 h. As shown in Figure 3, TNF-α increased the expression of LIF in the cytoplasm of nestin-positive cells. The co-localization of LIF with nestin suggests that LIF is indeed produced by human NPCs following TNF-α treatment.

### LIF is involved in TNF-α induced STAT3 activation and astrogliogenesis

**Figure 4:**
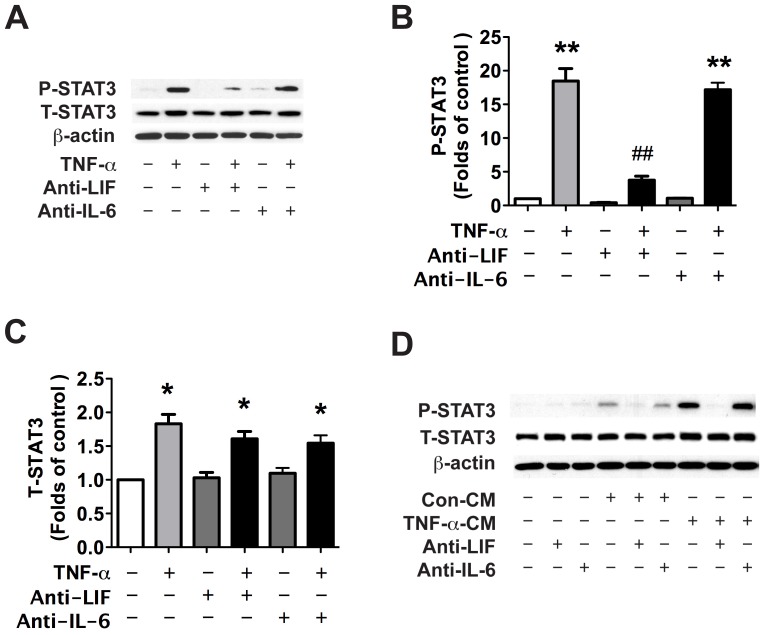
TNF-α induces STAT3 phosphorylation through the autocrine secretion of LIF. **A**. Human NPCs were pre-treated with neutralizing antibody for LIF or IL-6 (Anti-LIF or Anti-IL-6) for 1 h and then treated with TNF-α for 24 h. Expression of P-STAT3 and T-STAT3 were detected by Western blotting. β-actin was used as a loading control. **B–C**. The films were scanned and the acquired images were analyzed using the public domain NIH image program for data quantification. Expression of P-STAT3 (B) and T-STAT3 (C) were normalized to β-actin. Data is presented as fold of control expression. Results are average of three independent donors. * p<0.05, ** p<0.01 in comparison to control. ## p<0.01 in comparison to TNF-α treatment. **D**. Con-CM and TNF-α-CM were pre-incubated with neutralizing antibodies for LIF or IL-6 for 1 hour at 37°C. Cells were then treated with NCM with or without neutralizing antibodies for 30 min. Expression of P-STAT3 and T-STAT3 were detected by Western blotting. β-actin was used as a loading control. Results are representative of two independent experiments.

Because IL-6 and LIF were identified as the cytokines up-regulated by TNF-α stimulation in NPCs, we next studied their possible involvement in TNF-α-induced STAT3 activation and NPC differentiation. NPCs were pre-treated with neutralizing antibodies for LIF or IL-6 and then treated with TNF-α for 24 h. LIF neutralizing antibody, but not IL-6 neutralizing antibody, significantly inhibited TNF-α-induced STAT3 phosphorylation (Figure 4A, B). Notably, TNF-α also increased total STAT3 (T-STAT3) expression, which may aid the activation of STAT3 at the delayed time points.

To test whether the presence of LIF and/or IL-6 was responsible for TNF-α-CM-induced STAT3 activation, we pre-incubated CM with neutralizing antibodies for LIF or IL-6 for 1 h and then treated NPCs with CM for 30 minutes. LIF neutralizing antibody, not IL-6 neutralizing antibody, significantly attenuated TNF-α-CM-induced STAT3 phosphorylation (Figure 4D). Taken together, these results suggest that LIF, secreted from TNF-α-treated NPCs, is responsible for STAT3 activation following TNF-α.

**Figure 5:**
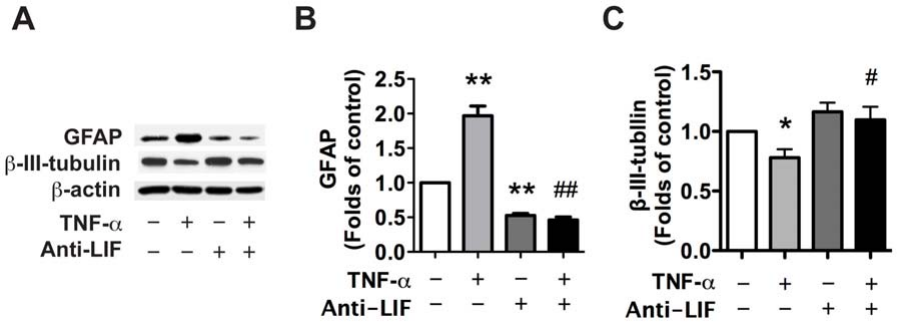
TNF-α induced astrogliogenesis through the autocrine secretion of LIF. **A**. Human NPCs were pre-treated with neutralizing antibody for LIF and were then treated with TNF-α for 6 d. Expression of GFAP, and β-III-tubulin were detected by Western Blot. **B–C**. The films were scanned and the acquired images were analyzed using the public domain NIH image program for data quantification. Expression of GFAP (B), and β-III-tubulin (C) were normalized to β-actin. * p<0.05, ** p<0.01 in comparison to control, while # p<0.05, ## p<0.01 in comparison to TNF-α treatment.

To test if LIF also contributes to TNF-α-induced astrogliogenesis, we differentiated NPCs with TNF-α in NB27 differentiation medium with or without LIF neutralizing antibody for 6 days. TNF-α significantly increased GFAP expression, a marker for astrocytes (Figure 5A, B) and inhibited β-III-tubulin expression, a marker for neurons (Figure 5A, C), suggesting an increase of astrogliogenesis and inhibition of neurogenesis. LIF neutralizing antibody attenuated TNF-α-induced astrogliogenesis (Figure 5A, B) and also reversed TNF-α-induced inhibition of neurogenesis (Figure 5A, C).

**Figure 6:**
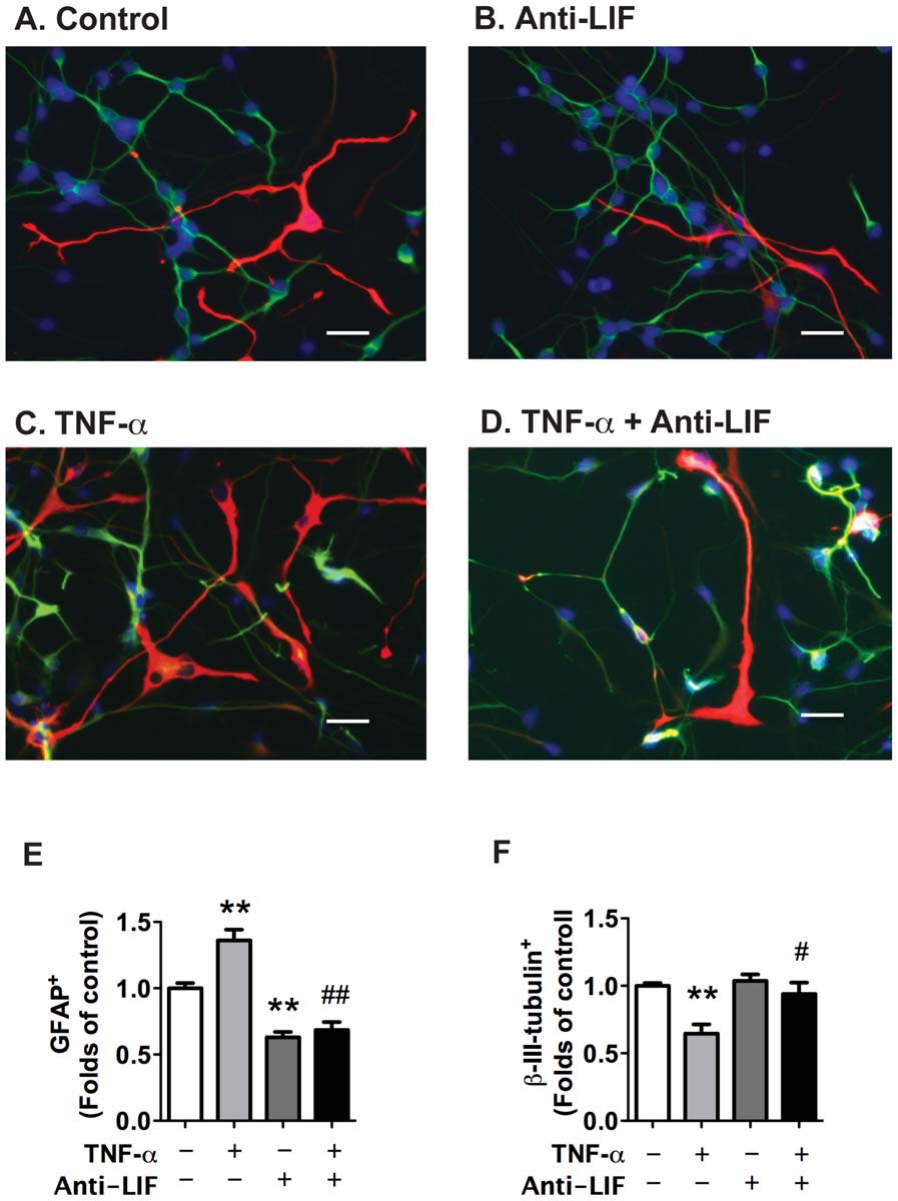
TNF-α-induced increase of astrocyte and decrease of neuronal proportions is through the autocrine secretion of LIF. Human NPCs were pre-treated with neutralizing antibody for LIF and were then treated with TNF-α for 6 d. **A–D**. Representative fluorescence overlay micrographs display the morphology of neurons (green) and astrocytes (red) in control, TNF-α, Anti-LIF, and TNF-α with Anti-LIF (TNF-α+Anti-LIF). Nuclei were stained with DAPI (blue). Original magnification is x 20 (scale bar 10 µm). **E–F**. GFAP (E) or β-III-tubulin (F) positive cells were quantified; data is presented as fold of control. Results are representative of two independent experiments. * p<0.05 in comparison to control, # p<0.05 in comparison to TNF-α.

To further evaluate the effect of LIF neutralizing antibody on TNF-α-induced astrogliogenesis, we used immunocytochemistry to visualize the change of GFAP-positive cells (Figure 6). TNF-α treatment significantly increased the proportion of the GFAP-positive cells and decreased the proportion of the β-III-tubulin-positive cells (Figure 6E, F). As expected, LIF neutralizing antibody significantly inhibited TNF-α-induced astrogliogenesis and partially abrogated TNF-α-induced inhibition of neurogenesis (Figure 6E, F). Taken together, these results suggest that TNF-α-induced astrogliogenesis is through the release of LIF in an autocrine manner.

## Discussion

Neural precursor cells, despite being multipotent, differentiate into astrocytes rather than neurons *in situ* during brain injury. The lack of significant neurogenesis in damaged brain areas may be due to the absence of molecules necessary for neuronal differentiation and/or the presence of molecules that favor the differentiation of NPCs toward other phenotypes. In pathological conditions of the CNS that are associated with neuroinflammation, activated resident immune cells (microglia and perivascular macrophages) produce a large number of proinflammatory cytokines and chemokines that affect the capacity of brain stem cells and alter neurogenesis [10], [15], [22]–[24]. Chronic brain inflammation has long been suspected to create detrimental and unfavorable conditions for neurogenesis. Despite this belief, little data is available for whether and how inflammatory factors regulate NPC differentiation. In the present study, we identify a unique mechanism of how TNF-α induces STAT3 activation and astrogliogenesis. Our observations demonstrated that factors released from NPCs, such as proinflammmatory cytokines IL-6 and LIF, could also contribute to the neuroinflammation, and affect cell fate determination through an autocrine manner.

Cell-fate determination during the differentiation of neural stem cells into specific neuronal and glial cell lineages is a highly orchestrated process. TNF-α has been shown to exert critical functions in survival, proliferation, and neuronal differentiation of NPCs, though the specific mechanisms through which TNF-α mediates these processes are not fully resolved due to conflicting results in the published literature [13], [14], [16]. TNF-α has been shown to negatively affect neurogenesis through compromising the survival of newly formed post-mitotic neurons [13], [15]. Keohane *et al.* also reported that TNF-α inhibited neuronal differentiation and increased astrocytic differentiation of hippocampal NPCs through increased expression of Hes1 [14]. In agreement with this observation, our previous studies using human fetal cortical NPC culture have suggested that TNF-α induced astrogliogenesis and inhibited neurogenesis via the Jak-STAT3 pathway [18].

The Jak-STAT3 signaling is a critical component of the astrogliogenic machinery during brain development [20], [25]. Our previous study demonstrated that STAT3 is involved in TNF-α-induced astrogliogenesis and inhibition of neurogenesis [18]. However, it is not likely that TNF-α is a direct upstream effector for STAT3 activation in human NPCs, as TNF-α treatment did not induce immediate phosphorylation of STAT3 (at 30 min). Instead, TNF-α activated STAT3 at delayed time points (6 h and 24 h) (Figure 1). Meanwhile, conditioned medium collected from TNF-α-treated NPCs induced immediate STAT3 activation at 30 min, but not at 6 h and 24 h, suggesting that TNF-α activates STAT3 indirectly through induction of upstream regulators of STAT3.

Members of the IL-6 cytokine family such as, LIF, IL-6 and CNTF, are able to activate the Jak-STAT signaling pathway and promote astroglial differentiation [20], [21]. We have detected IL-6 family cytokine expression in NPCs upon TNF-α treatment and found that TNF-α dramatically increased the mRNA expression of IL-6 and LIF but not CNTF (Figure 2). Using neutralizing antibodies, LIF was identified as the molecule responsible for the preferential differentiation of NPCs toward astrocytic lineage (Figure 4,5,6).

LIF signals through the heterodimeric complex of common glycoprotein 130 (gp130) and LIF receptor (LIFR) subunits. A number of studies have shown that gp130/LIFR-mediated signaling has pleiotropic action on different cell types. LIF is well known for promoting mouse embryonic stem (ES) cell self-renewal [26] and the maintenance/self-renewal of cultured mouse and human embryonic NSCs [27]–[31]. In addition, LIF-induced Jak-STAT signaling is critical for promoting astrocytic differentiation [20], [32], [33]. LIF is abundantly secreted from rat cortical neural precursor cells and serves as an autocrine/paracrine factor for the survival and astrocytic differentiation of embryonic cortical precursor cells [33]. In our study, we further demonstrated that TNF-α increased LIF production in human NPCs and LIF is responsible for TNF-α-induced STAT3 activation and the sequential astrogliogenesis.

In this study, we also observed the up-regulated expression of IL-6 in NPC. However, neutralizing antibody for IL-6 failed to block TNF-α-induced activation of STAT3, suggesting IL-6 may not be the main contributor for TNF-α-induced STAT3 activation and astrogliogenesis. This may be due to the lower expression of IL-6 receptor on NPCs (less than 1% of LIF receptor mRNA values, data not shown).

In conclusion, we have demonstrated that TNF-α activates STAT3 and promotes astrogliogenesis through the autocrine secretion of LIF. This study provides a novel mechanism by which pro-inflammatory cytokines affect neurogenesis and regulate the fate of NPCs. This information is important for developing transplantation therapies or promoting activation of endogenous NPCs for repair in neurodegenerative diseases with brain inflammation.
